# Health Promotion in the Workplace: Assessing Stress and Lifestyle With an Intranet Tool

**DOI:** 10.2196/jmir.1798

**Published:** 2011-11-08

**Authors:** Daniela Lucini, Nadia Solaro, Alessandro Lesma, Veronique Bernadette Gillet, Massimo Pagani

**Affiliations:** ^1^Centro di ricerca sulla Terapia Neurovegetativa e Medicina dell’EsercizioDipartimento Scienze ClinicheUniversità degli Studi di MilanoMilanItaly; ^2^Dipartimento di StatisticaUniversità degli Studi di Milano-BicoccaMilanItaly; ^3^ENI corporateMilanItaly; ^4^U.O. Telemedicina e Medicina dello SportOspedale “Luigi Sacco”MilanItaly

**Keywords:** Stress, lifestyle, risk factor, prevention, web-based questionnaire

## Abstract

**Background:**

Chronic noncommunicable conditions, particularly cardiovascular and metabolic diseases, are the major causes of death and morbidity in both industrialized and low- to middle-income countries. Recent epidemiological investigations suggest that management of lifestyle factors, such as stress and lack of physical activity, could have an important value in cardiometabolic conditions, while information technology tools could play a significant facilitatory role.

**Objectives:**

The objective of our study was to verify the feasibility of using a private website, directed to the workers of a major Italian company, to describe their health profile and lifestyle and work habits using an ad hoc self-administered questionnaire.

**Methods:**

We administered anonymous multiple choice Web-based questionnaires to 945 participants (683 completed the task) as part of an ongoing health promotion program in a multinational company. Qualitative and quantitative data were synthesized with nonlinear principal component analysis to construct indicators (ie, variables) for stress, control, and lifestyle domains. Considering in addition absenteeism, the Calinski-Harabasz statistic and cluster analysis jointly differentiated seven clusters, which displayed different distributions of standardized classification variables. The final step consisted in assessing the relationship of the resulting seven subject typologies with personal data, illnesses, and metabolic syndrome status, carried out for the most part with descriptive methods.

**Results:**

Statistical analyses singled out two not-overlapping domains of stress and control, as well as three not-overlapping domains of physical activity, smoking, and alcohol habits. The centroids of the seven clusters generated by the procedure were significantly (*P* < .001) different considering all possible 21 comparisons between couples of groups. Percentage distributions of variables describing personal information (gender, age group, work category, illness status, or metabolic syndrome) within participant typologies show some noteworthy findings: females, workers aged 35–44 years, junior white collar workers, and respondents reporting illness were more prevalent in the stress group than in the overall studied population; preclinical metabolic syndrome status was more prevalent in the group with higher alcohol consumption. Absentees reported more illness.

**Conclusions:**

The present Intranet-based study shows the potential of applying diverse statistical techniques to deal jointly with qualitative and quantitative self-reported data. The resulting formal description of subject typologies and their relationship with personal characteristics might provide a convenient tool for supporting health promotion in the work environment.

## Introduction

### Background

Chronic noncommunicable conditions, particularly cardiovascular and metabolic diseases, are the major causes of death and morbidity around the world, affecting both industrialized and low- to middle-income countries. Recent epidemiological investigations [1,2] showed that lifestyle factors, such as stress and lack of physical activity, provide additional prognostic information to that furnished by usual coronary risk factors, suggesting that their management might have clinical value [3-5]. Behavioral components of risk are, in addition, difficult to handle because they cannot be treated by traditional pharmacological means and require the active collaboration of patients, who must change their attitudes and habits [6-9]. Lifestyle components, such as stress and exercise, have the advantage of being assessable individually with information technology (IT)-administered questionnaires [10], although their self-reported nature mandates additional caution in interpreting findings [11]. Stress can be described by various personal (symptom profile, psychological distress, and fatigue perception), social (family and work environment) [12], and functional domains (autonomic and hormonal regulation) [12-16]. Physical exercise can be defined in terms of intensity, modality (strength or aerobic training), and duration, leading to an algorithmic evaluation of workout level in a given time period [9,17,18].

Maintaining an ideal health risk profile in middle age might have important implications for greater longevity, compression of disease, increased quality of life, and reduced costs [19]. Because only a very limited fraction of the population (about 5%) fits into the ideal risk limits, new techniques must be tested to reach these new goals; these techniques might encompass the introduction of Web applications [20] with a focus on lifestyle [21]. We have been testing a behavioral approach to cardiovascular prevention, focusing on stress and inactivity in addition to usual risk factors, in various settings ranging from the outpatient clinic [15] to the workplace [13]. We also tested the feasibility of simple IT applications for technician-mediated [22] or self-reported [10] data entry. The use of Web- (or Intranet-) based approaches might also be suitable to deliver essential training with digital techniques and minimal cost [22-26], accommodating any personal preference for site, time, or pace, possibly also furnishing useful clinical feedback, whereby congruent multiparameter models, such as metabolic syndrome (MeS) [27], might be easier to handle [22].

### Aims

With this in mind we designed this exploratory investigation to verify the feasibility of using an Intranet-based tool in the workplace [10] intended to assist employees of an Italian company to optimize lifestyle and cardiometabolic risk, as part of a company’s health promotion initiative. Specific constraints were strict anonymity, minimal investments, and specific targets of physical activity, eating habits, and stress, with adherence to the company’s privacy policy. In this report we present a methodology to describe the baseline status of a group of employees who agreed to participate in this initial exploratory phase of the study. Metabolic risk was approximated by using the MeS model, according to the Adult Treatment Panel III (ATP III) definition [27].

## Methods

This study is part of an ongoing Web-based health promotion initiative of a major Italian multinational company. At this initial stage of the project, through the company Intranet, workers were offered an information service on health promotion, focusing on various work- and non-work-related issues, ranging from influenza epidemics to healthy lifestyle. In addition, as a company benefit, workers could log on to the health portal and enroll in an educational project based on a self-administered Web questionnaire [10], eventually aiming at optimizing lifestyle and minimizing cardiometabolic risk. According to the company’s policy, the project required strict anonymity that was guaranteed by the use of name and password protection chosen by participants and maintained secret.

### The Health Questionnaire

The anonymous questionnaire, which is an extended project-tailored version of the Subjective Stress Symptoms Scores Questionnaire (4SQ; previously described [13-16]), contains 50 multiple choice questions, addressing various domains related to working conditions (job level and absenteeism), living and exercise habits, and perceived stress and control. In addition, participants were asked to gather (if available) their most recent (<3 months old) reports on blood chemistry (total and high-density lipoprotein cholesterol, triglycerides, glucose), blood pressure, heart rate, and anthropometrics (including waist circumference), and to enter these numerical values. Detailed instructions about compilation were given through a short movie tutorial available through the company’s portal. 

The number of questions was a balance between the time required to fill out the questionnaire and the detail of the inferences that could be drawn.

Weekly activity levels were estimated from the approximate daily amount (in minutes) and nominal intensity, and expressed in (estimated) metabolic equivalents (METs), using a validated approach [17,18].

Psychological distress was estimated from the following items: bodily symptom perception, stress perception, fatigue perception, and control perception, as in our previous studies on this topic [13-16]. After answering the questionnaire, participants were provided with a graphical answer that illustrated potential areas of lifestyle improvement [10]. If participants subsequently wished to verify any changes possibly related to effects of lifestyle interventions, they were allowed to use the questionnaire again. The present study addressed only the initial descriptive part.

### Participants

Participation in the study was under the guarantee of strict anonymity, and the questionnaire was made available for a month, from October to November 2008. At the time of the study about 24,000 workers of the Italian branch of the company had access to the Intranet as a fundamental instrument for everyday work. About 9970 workers accessed the complementary health portal every month; of these 4877 read information regarding the ongoing preventive campaign, 1380 saw the detailed instructions to fill out the questionnaire, and 945 employees elected to actively participate in the survey, on a completely voluntary basis. Employees were motivated to accurately fill out the questionnaire by the possibility of immediately obtaining an individual map of their risk factor profile and areas for improvement based on input data [10]. 

To optimize data quality, we excluded those participants who did not complete their reports or who provided unrealistic data, particularly regarding biochemistry, blood pressure, or anthropometrics. The final data set comprised 683 participants.

The protocol of the study was approved by the Institutional Ethics Committee, as part of an ongoing investigation on lifestyle-based prevention.

### Statistics and Data Handling

**Figure 1:**
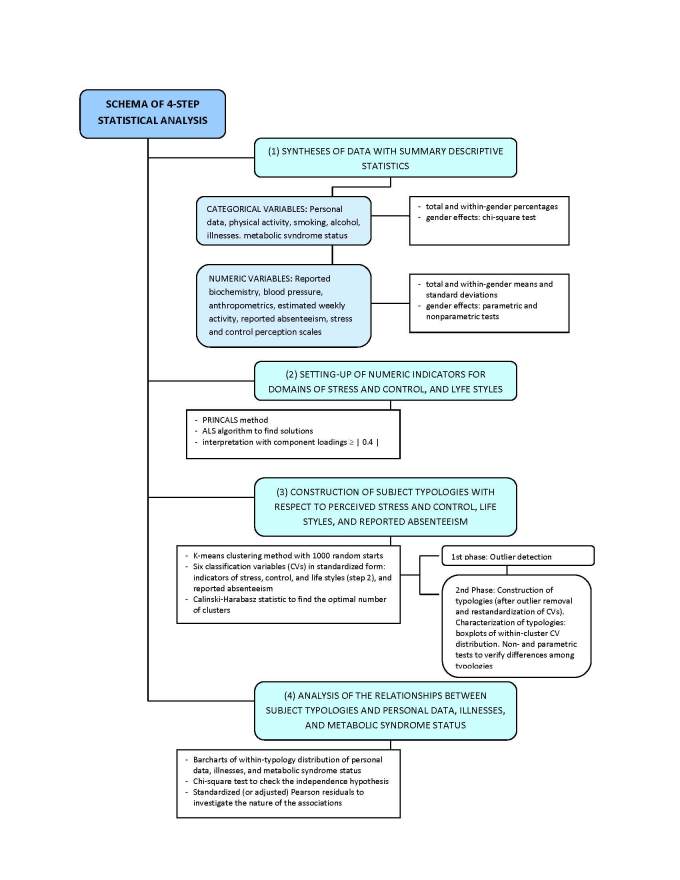
Schematic outline of the phases of data analysis (ALS = alternating least squares, PRINCALS = nonlinear principal component analysis).

The main goal of disclosing and assessing possible relations linking cardiometabolic risk factors with perceived stress and control and with lifestyle is accomplished in four key steps (Figure 1): (1) synthesizing the information collected by the health questionnaire with summary descriptive statistics, (2) setting up quantitative indicators for perceived stress and control and for lifestyles, (3) building respondents’ typologies with respect to perceived stress and control, lifestyles, and reported absenteeism, (4) assessing the presence of relationships between respondents’ typologies and their personal data, illness status, and the MeS [27]. We mostly performed statistical analyses according to a data-driven approach by using exploratory multivariate data analysis techniques—that is, the nonlinear principal component analysis (PRINCALS) method [28] and k-means clustering algorithm [29]. We also performed statistical tests, although in this investigation inferences should have a minor role. The target population, to which inferences should be referred, was not clearly identified due to respondents’ self-selection, thus suggesting some caution in our interpretations. We applied both parametric and nonparametric testing procedures [29,30] to take advantage of their specific potentials and to overcome their specific limits. We considered a test result to be “sufficiently revealing” if it was borne out as significant by both procedures.

With specific reference to the first step of analysis, summary descriptive statistics are presented in Table 1 and Table 2. The fourth column in Table 1 reports percentage distributions of personal data, lifestyle, and illness variables for the 683 participants in the study. To show the extent of gender differences, within-gender percentage distributions—percentages computed for each variable given (or conditionally to) the gender—are also provided in different columns. We performed a chi-square test to verify whether the above variables and gender could be assumed to be statistically independent (last column of Table 1). 

Table 2 presents means and standard deviations of variables pertaining to reported biochemistry and anthropometrics, estimated weekly activity, reported absenteeism, and perceived stress and control scales. We also computed summary statistics for males and females separately. The presence of significant gender effects was assessed through both parametric univariate analysis of variance (ANOVA) and nonparametric Mann-Whitney and 2-sample Kolmogorov-Smirnov testing procedures. The (null) hypotheses to check for each variable were the equality of within-gender means (ANOVA) and the equality of the two within-gender distributions (Mann-Whitney and Kolmogorov-Smirnov).

**Table 1:** Distribution of data (N = 683 participants): total and within-gender percentages

Variable					Male	Female	Total	*P* value^a^
**Personal data**								
	Gender				495/683, 72.5%	188/683, 27.5%		
	Work category							<.001
			Blue collar		24/495, 4.8%	1/188, 0.5%	25/683, 3.7%	
			Junior white collar		249/495, 50.3%	124/188, 66.0%	373/683, 54.6%	
			Senior white collar		197/495, 39.8%	59/188, 31.4%	256/683, 37.5%	
			Manager		25/495, 5.1%	4/188, 2.1%	29/683, 4.2%	
	Age group (years)							ns^b^
			<35		55/495, 11.1%	26/188, 13.8%	81/683, 11.9%	
			35–44		133/495, 26.9%	58/188, 30.9%	191/683, 28.0%	
			45–54		234/495, 47.3%	87/188, 46.3%	321/683, 47.0%	
			>54		73/495, 14.7%	17/188, 9.0%	90/683, 13.2%	
**Lifestyle**								
	Smoking habit							ns^b^
			Nonsmoker		341/495, 68.9%	140/188, 74.5%	481/683, 70.4%	
			Quit >1 year		58/495, 11.7%	20/188, 10.6%	78/683, 11.4%	
			Quit <1 year		10/495, 2.0%	5/188, 2.7%	15/683, 2.2%	
			≤5 cigarettes/day		22/495, 4.4%	8/188, 4.3%	30/683, 4.4%	
			>5 and ≤10 /day		26/495, 5.3%	9/188, 4.8%	35/683, 5.1%	
			>10 and ≤20/day		29/495, 5.9%	5/188, 2.7%	34/683, 5.0%	
			>20/day		9/495, 1.8%	1/188, 0.5%	10/683, 1.5%	
	Intend to quit							ns^b^
			Yes, now		40/495, 8.1%	7/188, 3.7%	47/683, 6.9%	
			Yes, in 6 months		24/495, 4.8%	8/188, 4.3%	32/683, 4.7%	
			Does not intend to quit		30/495, 6.1%	8/188, 4.3%	38/683, 5.6%	
	Structured physical activity							ns^b^
			None		70/495, 14.1%	24/188, 12.8%	94/683, 13.8%	
			No, but would like to		133/495, 26.9%	43/188, 22.9%	176/683, 25.8%	
			Sometimes		51/495, 10.3%	21/188, 11.2%	72/683, 10.5%	
			About 1 hour/week		64/495, 12.9%	32/188, 17.0%	96/683, 14.1%	
			≤30 minutes/day, 3 times/week		81/495, 16.4%,	42/188, 22.3%	123/683, 18%	
			≤30 minutes/day, 5 times/week moderate activity or ≤20 minutes/day, 3 times/week vigorous activity		60/495, 12.1%	16/188, 8.5%	76/683, 11.1%	
			≤30 minutes/day every day moderate or intense activity		36/495, 7.3%	10/188, 5.3%	46/683, 6.7%	

	Wine or beer (glasses/week)							<.001
			None		72/495, 14.5%	78/188, 41.5%	150/683, 22%	
			1–2		147/495, 29.7%	77/188, 41.7%	224/683, 32.8%	
			3–7		169/495, 34.1%	21/188, 11.2%	190/683, 27.8%	
			8–14		77/495, 15.6%	8/188, 4.3%	85/683, 12.4%	
			15–21		20/495, 4.0%	3/188, 1.6%	23/683, 3.4%	
			22–30		7/495, 1.4%	0/188, 0%	7/683, 1.0%	
			>30		3/495, 0.6%	1/188, 0.5%	4/683, 0.6%	
	Alcohol (glasses/week)							<.001
			None		361/495, 72.9%	178/188, 94.7%	539/683, 78.9%	
			1–2		118/495, 23.8%	9/188, 4.8%	127/683, 18.6%	
			3–7		15/495, 3.0%	0/188, 0.0%	15/683, 2.2%	
			8–14		1/495, 0.2%	1/188, 0.5%	2/683, 0.3%	
**Illnesses**								ns^b^
		None			316/495, 63.8%	116/188, 61.7%	432/683, 63.3%	
		Functional illness			51/495, 10.3%	31/188, 16.5%	82/683, 12%	
		Organic illness			128/495, 25.9%	41/188, 21.8%	169/683, 24.7%	
**Metabolic syndrome status^c^**								<.001
		Normal			55/495, 11.1%	140/188, 74.5%	195/683, 28.6%	
		Preclinical			285/495, 57.6%	46/188, 24.5%	331/683, 48.5%	
		Metabolic syndrome			155/495, 31.3%	2/188, 1.1%	157/683, 23.0%	

^a^ Significance level in the chi-square test for testing the null hypothesis of independence of variables and gender.

^b^ Not significant (*P* > .05).

^c^ Metabolic syndrome is inferred from data presented in Table 2.

**Table 2:** Descriptive data (N = 683 participants)^a^

Variables		Total		Male		Female		Reference values
		Mean	SD	Mean	SD	Mean	SD	
**Reported biochemistry, blood pressure, and anthropometrics**								
	Total cholesterol (mg/dL)	203.23	38.03	203.92	38.76	201.43	36.07	<200
	HDL^b^ cholesterol (mg/dL)**^,^^††^^,^^‡‡^	60.01	23.37	56.68	23.13	68.79	21.70	Male: >29, female: >35
	LDL^c^ cholesterol (mg/dL)*^,^^†^^,^^‡‡^ (Friedewald formula)	120.48	40.08	122.61	41.49	114.86	35.59	<100
	Triglycerides (mg/dL)**^,^^††^^,^^‡‡^	113.72	71.03	123.16	74.44	88.88	53.91	<150
	Glucose (mg/dL)**^,^^††^^,^^‡‡^	90.09	16.63	91.89	17.79	85.34	11.90	74–106
	Systolic blood pressure (mmHg)**^,^^††^^,^^‡‡^	122.73	11.23	124.51	10.17	118.06	12.50	<120
	Diastolic blood pressure (mmHg)**^,^^††^^,^^‡‡^	78.54	7.91	79.44	7.20	76.15	9.12	<80
	Heart rate (beats/minute)**^,^^††^^,^^‡^	70.09	10.12	69.22	10.15	72.37	9.70	60–90
	Weight (kg)**^,^^††^^,^^‡‡^	75.27	13.60	79.72	11.57	63.55	11.43	NA^d^
	Height (cm)**^,^^††^^,^^‡‡^	172.93	7.86	176.02	6.00	164.78	6.18	NA
	Body mass index (kg/m^2^)**^,^^††^^,^^‡‡^	25.06	3.61	25.71	3.39	23.35	3.62	<25
	Waist circumference (cm)**^,^^††^^,^^‡‡^	90.42	12.06	93.76	10.30	81.63	11.98	Male: <102, female: <88
**Estimated weekly activity (metabolic equivalents, minutes/week)**								
	Walking	436.17	451.37	455.98	469.64	384.03	395.75	
	Moderate activity	378.38	445.24	370.26	449.14	399.73	435.27	
	Vigorous activity**^,^^††^^,^^‡‡^	551.59	822.88	630.80	857.98	343.02	681.59	
	Total activity**^,^^†^^,^^‡^	1366.14	1239.34	1457.05	1275.36	1126.78	1106.97	
**Reported absenteeism**								
	Lost working days (in previous 12 months)^††^^,^^‡‡^	5.87	14.80	5.34	16.29	7.25	9.74	
**Stress and control perception scales (AU)^e^**								
	4SQ^f^ **^,^^††^^,^^‡‡^	16.86	20.11	14.75	18.53	22.41	22.91	
	Stress**^,^^††^^,^^‡‡^	2.64	2.70	2.53	2.72	3.67	3.05	
	Fatigue**^,^^††^^,^^‡‡^	2.84	2.86	2.28	2.54	3.60	2.88	
	Control	4.11	3.16	4.17	3.27	3.95	2.83	

^a^ Although in the subsequent steps of analysis statistical evaluation of perceived stress and control scales is performed in nonmetric terms, in this table, for practical reasons, they are presented as means and SD.

^b^ High-density lipoprotein.

^c^ Low-density lipoprotein.

^d^ Not applicable.

^e^ Arbitrary units.

^f^ Subjective Stress Symptoms Score Questionnaire.

Significance level in the univariate analysis of variance (the null hypothesis is the equality of within-gender means): *significant at the .05 level, **significant at the .001 level. Actual *P* value for LDL cholesterol is *P* = .02.

Significance level in Mann-Whitney test (the null hypothesis is the equality of within-gender distributions): ^†^significant at the .05 level, ^††^significant at the .001 level. Actual *P* value for LDL cholesterol is *P* = .01.

Significance level in Kolmogorov-Smirnov test (the null hypothesis is the equality of within-gender distributions): ^‡^significant at the .05 level, ^‡‡^significant at the .001 level. Actual *P* value for LDL cholesterol is *P* = .03; for heart rate is *P* = .01; for total activity is *P* = .01.

Regarding step (2), we set up numeric indicators to represent the underlying domains of stress, control, and lifestyles, with the categorical principal component analysis method (CATPCA, SPSS version 18; IBM Corporation, Somers, NY, USA), also known in the statistical literature as nonlinear principal component analysis, or PRINCALS [28]. PRINCALS is an advanced multivariate statistical analysis technique addressed to data dimensionality reduction problems. It is still relatively little used in many fields of application, probably due to its intrinsic complexity, but it offers potentials in data analysis not shared by ordinary methods. Since it is not so well known yet, we provide a brief description of its main characteristics.

Unlike other methods such as principal component analysis (PCA) or factor analysis, PRINCALS can jointly handle qualitative and quantitative variables to convey their informational content in a small, a priori fixed number of dimensions (ie, unobservable variables), thus synthesizing data with the least possible loss of information. PRINCALS constructs dimensions that have zero mean and are pairwise uncorrelated by minimizing a loss function under several restrictions [28]. The minimization is made with respect to a set of unknown quantities, namely object (or dimension) scores (ie, values of dimensions) and category quantifications (ie, values that are attributed to categories of the original qualitative variables). Unlike PCA, the minimization problem does not admit a closed-form solution. It requires iterative procedures (Figure 1), specifically the alternating least squares algorithm. This is essentially a 2-step process that alternates updated solutions over object scores and category quantifications. Within the PRINCALS routine, qualitative variables are quantified, or optimally scaled, in the sense that their categories are replaced by metric values. So, optimal scaling transformations account for the different measurement level of variables. Nominal variables are quantified through application of the so-called centroid principle [28]. Ordinal variables are quantified through the weighted monotonic regression transformation, which allows the rank order to be preserved among ordinal categories. Numerical variables, being already metric, are generally simply standardized. More complex transformations can also be involved—for example, spline functions could be applied to nominal and ordinal variables [28]. Summing up, PRINCALS reaches two goals simultaneously: quantifying where necessary, and reducing the number of original variables (both qualitative and quantitative) by extracting dimensions. In addition, the PRINCALS routine automatically rotates the extracted dimensions to refer them to their principal axes in analogy with PCA. Accordingly, the computed dimensions reproduce the maximum possible variation in data or, more precisely, in optimally scaled variables.

After extraction, dimensions, being unobservable variables, require interpretation in order to establish which semantic fields or domains of original variables they account for. Interpretation is mainly based on the so-called component loadings, which are linear correlation coefficients of dimensions and optimally scaled variables. Dimensions assume the meaning from the variables with which they are more highly correlated, in a negative or positive sense. Usually, a threshold (absolute) value of 0.4 is introduced to distinguish negligible (<|0.4|) from essential (≥|0.4|) component loadings. Once their meaning is assessed, dimensions are likely to be treated as indicators of the specific semantic fields they represent.

Another aspect of concern regards the choice of the “ideal” number of dimensions to be extracted. Such a number has to be fixed before the PRINCALS routine starts. In this study we tackled this problem by relying on a combination of several criteria: parsimony (few dimensions give a simpler description of data), accuracy (many dimensions fit data better), and interpretability (dimensions accounting for smaller proportions of variance tend to explain noise in data, rather than a systematic tendency). Accuracy is assessed through total and per-variable variance accounted for (VAF) indices, which provide the percentage of variance relating to the set of optimally scaled variables that is accounted for by the whole set of extracted dimensions (total VAF) and the single dimensions taken one at a time (per-variable VAF). The Cronbach alpha index [31] is also provided. It assesses the degree of internal consistency of (optimally scaled) variables and their relating indicator, to verify whether they jointly measure the same construct. This further supports the interpretations.

In this way, by applying PRINCALS to the set formed by the 4SQ scale and the perceived stress, control, and fatigue scales (ordinal data) [13-16], we obtained stress and control (numeric) indicators. As for lifestyle components, we obtained activity, smoking, and alcohol (numeric) indicators by applying PRINCALS to the whole set formed by the lifestyle qualitative variables (Table 1) and the quantitative estimation in METs of activity (Table 2).

Subsequently, with regard to step (3) (Figure 1), we employed the k-means clustering method [29] to construct subject typologies with respect to the following six components used as classification variables: stress and control indicators, lifestyle indicators (ie, activity, smoking, alcohol), and reported absenteeism (Table 2). The k-means clustering method is a nonhierarchical, iterative algorithm of classification that forms clusters (or groups) by minimizing the (squared) Euclidean distance between subjects and cluster centroids—that is, within-cluster vectors containing the means of variables. This is the same as forming clusters by minimizing the within-cluster deviance (ie, sum of squares).

This method has some well-known weaknesses: (1) final classification may depend on the order in which subjects appear in the data matrix, and (2) the number *k* of groups has to be fixed a priori. We addressed the problem of order dependency (problem 1) by employing a k-means cluster with *random starts*. A random start implies that the algorithm is initialized by choosing the *k* subjects, which have to represent the initial *k* clusters (so-called *seeds*), at random and without replacement. Then, the procedure iteratively attributes the remaining subjects to the nearest cluster on the basis of the squared Euclidean distance computed after updating the cluster centroids. Usually, it is advisable to adopt a great amount of random starts, and choose the partition that guarantees the minimum within-cluster deviance, so as to form groups that are as homogeneous as possible. Regarding problem (2), how to choose the ideal number of groups, we compute the Calinski-Harabasz (CH) statistic [32], which is given by the ratio of between-cluster variance to within-cluster variance. Both these variances derive from the division of the corresponding deviances by degrees of freedom equal to *k* in between-cluster variance and *N* – *k* in within-cluster variance, where *N* is the total number of subjects. Such a statistic is thus adjusted for the number of groups, and results derived from different classification schemes can be directly compared. The larger the statistic value, the greater the separation between groups, and the better the classification scheme pertaining to that specific partition in *k* groups.

An interesting feature of k-means clustering is its capacity to detect outliers—subjects with anomalous features with respect to the majority of data. If the algorithm is carried out as the number of groups increases, it may reveal small groups of isolated subjects that stably remain the same from a specific *k* onward. These small clusters can then be regarded as individuals or groups of outlying units, which can be removed and handled separately if proven to strongly affect results. 

In this study, we performed k-means clustering (Figure 1) with 1000 random starts with the number of groups varying from *k* = 2 to *k* = 15 in two different phases. In the first phase, the algorithm was run with the specific goal of detecting potential outliers. To guarantee the same weight in the classification process, all six classification variables were standardized (*z* score) to have a mean of 0 (SD 1) before entering the clustering procedure. This shows the presence of six outliers (0.9% of the population), five falling in the same cluster plus one being isolated, which we therefore discarded in subsequent analyses. In the second phase, after removal of outliers, classification variables were standardized again. The algorithm was then performed as before on the remaining 677 participants. According to the CH statistic, seven is the optimal number of groups. Interpretation of clusters as subject typologies was carried out by means of boxplots of the within-clusters distribution of the classification variables. Typologies were labeled by the prevailing aspects that distinguished them from each other. In the absence of benchmarking and within the constraints of the present preliminary study, validation of groups was appraised with inferential procedures. Significance of differences between clusters was assessed with both parametric (univariate and multivariate ANOVA [MANOVA], and squared Mahalanobis distance test) and nonparametric (Kruskal-Wallis test) testing procedures [29,30], thus allowing for evaluation of the importance of all classification variables simultaneously, as well as one variable at a time. In particular, parametric procedures verified the hypotheses of equality of all cluster means for each single variable (ANOVA), equality of all cluster centroids (MANOVA), and equality of cluster centroids compared pairwise (squared Mahalanobis distance test). Kruskal-Wallis test, the nonparametric version of ANOVA, allows the equality of all cluster medians for each single variable to be checked.

Regarding step (4) (Figure 1), we first inferred the MeS status by the presence of at least three of the following ATP III criteria: (1) blood pressure ≥130/85 mmHg, (2) triglyceride levels ³150 mg/dL, (3) high-density lipoprotein cholesterol <40 mg/dL for males and <50 mg/dL for females, (4) fasting glycemia >100 mg/dL, and (5) waist circumference >102 cm for males and >88 cm for females. Participants with fewer than three factors were classed as preclinical, and those with no factors were classed as normal (Table 1).

Subsequently, we studied personal data (gender, work categories, and age group), illness, and MeS status with respect to subject typologies by computing within-typology and total percentages. Total percentages are computed for each variable on the whole set of participants without considering their aggregation in typologies. They can also be viewed as weighted arithmetic means of their corresponding within-typology percentages, where weights are given by the number of participants falling in the corresponding categories of the variables. Comparisons between total and within-typology percentages therefore reveal whether certain participants’ characteristics tend to concentrate more highly (or more mildly) in specific groups than in the population. This analysis is mostly carried out in descriptive terms, by means of barcharts of within-typology and total percentage distributions computed for each variable. An overall chi-square test is first performed to verify whether subject typologies and those variables, considered one at a time, are statistically independent. 

Subsequently, to learn more about the nature of associations between subject typologies and the various categories on personal data, illnesses, and MeS status, as revealed by chi-square tests, we computed standardized (or adjusted) Pearson residuals (APRs) [33]. For each 2-way contingency table obtained by cross-classifying subjects with respect to typologies and the above variables, APRs are given by the differences between corresponding observed and expected (ie, under the independence hypothesis) frequencies of subjects, which are then divided by their standard errors [33]. Given that APRs are asymptotically standard normal, inferences can be drawn, and significant single associations between typologies and categories of the above variables can be detected. Usually, an APR is considered “too great” to be consistent with a no-association hypothesis if it exceeds 2 or 3 in absolute value. Expressed in terms of (2-tailed) *P* values (taken from the standard normal), the two thresholds correspond, respectively, to *P* = .046 and *P* = .003. In this study, we introduced the value of 3.5 as well, which allows significant results to be detected at the level of *P* < .001.

Unless otherwise indicated, throughout this study the significance level was set at the .05 level. PRINCALS analysis, ANOVA, MANOVA, Kruskal-Wallis, Mann-Whitney, Kolmogorov-Smirnov, chi-square test, and APR were performed with SPSS version 18 (IBM Corporation, Somers, NY, USA); k-means clustering with random starts and graphics appearing next were carried out in the R environment, version 2.13.0 (R Foundation, Vienna, Austria); the squared Mahalanobis distance test was computed with SAS version 9.1 (SAS Institute, Cary, NC, USA).

## Results

Below we present results of analyses following the four steps schematized in the Statistics section (Figure 1).

### Summary Descriptive Statistics

Descriptive personal and lifestyle data of 683 participants are presented in Table 1.

Their modal age was 45–54 years, and gender was 72.5% (495/683) male and 27.5% (188/683) female. They were in large majority (629/683, 92.1%) white collar workers (only 3.7%, 25/683, blue collar workers and 4.2%, 29/683, managers) and were almost solely from one country (Italy). The majority of females (124/188, 66% females) were junior white collar workers, while a higher proportion of males than females occupied blue collar (24/495, 4.8% males vs 1/188, 0.5% females), senior white collar (197/495, 39.8% males, vs 59/188, 31.4% females), and management positions (25/495, 5.1% males, vs 4/188, 2.1% females).

Data show that age was unevenly distributed, although the distributions of males and females over age groups are similar. More than 70% (481/683) participants were nonsmokers, in accord with the smoking ban of the company. Almost 40% (270/683) declared an absence of physical activity; the majority (374/683, 54.8%) did not drink or remained within 1 to 2 glasses of wine or beer per week. Almost 80% (539/683) did not drink any alcohol, especially females (178/188, 94.7% females).

About 12% (82/683) of respondents said they had functional disturbances, and almost 25% (169/683) reported some form of chronic disease; the majority, however (432/683, 63%), said they had no active diseases. There were no apparent differences between males and females.

Almost 49% (331/683) of participants (preclinical) reported one or two risk factors for MeS, and 23% (157/683) had MeS status. Most females (140/188, 74.5%) declared no risk factors, while the majority of males appeared preclinical (285/495, 57.6%) or within MeS (155/495, 31.3%). Chi-square test showed significant differences between males and females as regards work categories, alcohol habit (wine or beer, and alcohol in glasses per week), and MeS status (see Table 1).

Table 2 summarizes statistics on reported biochemical and anthropometric data, corroborating that, as expected from a working population prevalently comprising middle-aged workers, mean population values are reasonably within or near normal limits. Estimated weekly METs of activity were quite low owing to substantial lack of moderate or vigorous activity in a large fraction (about 25%, first quartile) of the study population. Regarding reported absenteeism, yearly lost days were overall very low (mean 5.87 days/year) with a single notable exception (300 days). Average indices of perceived bodily symptoms, stress, fatigue, and control were all within the range that can be observed in normal individuals in our laboratory.

Parametric and nonparametric testing procedures (see Table 2) agree on supporting significant differences between males and females in all reported biochemistry (except total cholesterol), as well as blood pressure and anthropometrics. Vigorous and total activity, reported absenteeism, and 4SQ, perceived stress, and fatigue scales were also significantly different.

### Setting Up of Stress, Control, and Lifestyle Indicators

To account for the multivariate nature of domains under study, we applied the PRINCALS method to the two sets formed by (1) the four stress and control perception scales, and (2) lifestyle variables, thus obtaining, respectively (1) two dimensions, which can be interpreted as indicators of stress and control (total VAF: 84.2% of variance of the four optimally transformed scales of self-reported stress and control; total Cronbach alpha = .937) and (2) three dimensions interpreting lifestyles, which can be regarded as indicators of activity, smoking, and alcohol habit (total VAF: 70.1% of all optimally transformed lifestyle variables; total Cronbach alpha = .947).

These interpretations of the indicators derive from the analysis of component loadings. In particular, component loadings for the stress indicator are .885 with the 4SQ scale, .885 with the perceived fatigue scale, .870 with the perceived stress scale, and .310 with the perceived control scale. This denotes a strong linear relationship of the stress indicator with the first three scales, while the link with the control domain turns out to be negligible. Notably, the stress indicator accounts for 60.5% of the total variance. Moreover, Cronbach alpha computed for the (optimally transformed) 4SQ, perceived stress, and fatigue scales is equal to .934 if the stress indicator is involved in computations, but .865 if the stress indicator is disregarded. The three scales and their indicators are therefore characterized by a high internal consistency.

On the other hand, the component loadings for the control indicator are equal to –.033 with the 4SQ scale, –.172 with the perceived fatigue scale, –.129 with the perceived stress scale, and .949 with the perceived control scale. This suggests interpreting the second dimension in terms of the control indicator, which accounts for 23.7% of the total variance.

Interpretation of lifestyle dimensions as indicators is based on the component loadings reported in Table 3. It is apparent that dimension 1 is highly positively correlated with activity variables (VAF: 32.4%), dimension 2 with smoking variables (VAF: 21. 9%), and dimension 3 with alcohol variables (VAF: 15.8%). Moreover, Cronbach alpha values computed for each subset of the lifestyle variables (denoted with a, b, and c in Table 3) and the corresponding indicator are high (Table 3, next to last row), thus proving high internal consistency in all cases.

**Table 3:** Component loadings for lifestyle indicators

Lifestyle (quantified) variables		Lifestyle indicators		
		Dimension 1^a^	Dimension 2^b^	Dimension 3^c^
Smoking habit		–.067	.961^b^	–.200
Intend to quit		–.079	.958^b^	–.205
**Estimated metabolic equivalents of activity**				
	Walking	.571^a^	.102	.078
	Moderate activity	.710^a^	–.029	–.085
	Vigorous activity	.773^a^	.043	–.040
	Total activity	.975^a^	.057	–.032
Frequency of structured physical activity		.716^a^	–.032	–.112
Wine or beer (glasses/week)		.094	.202	.816^c^
Alcohol (glasses/week)		.065	.265	.802^c^
Variance accounted for		32.4%	21.9%	15.8%
**Cronbach alpha**				
	With dimension	.882	.983	.868
	Without dimension	.808	.967	.615

^a,b,c^ Component loadings with absolute value >0.4. a: dimension 1 = activity indicator, b: dimension 2 = smoking indicator, c: dimension 3 = alcohol indicator.

### Construction of Subject Typologies

The k-means clustering method with 1000 random starts was employed to form clusters of participants according to their scores on the indicators of stress, control, activity, smoking, and alcohol, and their reported absenteeism, all used as classification variables in standardized form. Table 4 reports their minimum and maximum values and quartiles. By comparing the maximum value with the third quartile, it is apparent that outlying participants (in a univariate sense) are present in the dataset. For instance, regarding reported absenteeism, 75% of participants had *z* scores equal at most to 0.0091, while the maximum value is 19.88. This clearly denotes the presence of at least one outlier. A similar argument can be advanced for the alcohol indicator and, though less apparently, also for the stress, activity, and smoking indicators. 

**Table 4:** Descriptive data of classification variables (N = 683 participants)

Indicators (*z* score)	Minimum	Maximum	1st quartile	2nd quartile	3rd quartile
Stress	–1.2404	3.3452	–0.7531	–0.3071	0.5719
Control	–2.1358	1.4964	–1.0103	0.0668	1.0068
Activity	–1.3373	4.6326	–0.7692	–0.2112	0.5596
Smoking	–0.6954	4.5797	–0.5658	–0.4533	–0.1202
Alcohol	–1.7381	10.6010	–0.5668	–0.2588	0.4445
Reported absenteeism	–0.3964	19.8797	–0.3964	–0.1937	0.0091

Moreover, standardized classification variables (Table 4) proved overall to have very low linear correlations (table omitted for simplicity), so no multicollinearity problem arose. This excludes the drawback of more highly correlated variables exerting higher weights on the classification procedure. By construction the three indicators of lifestyles are uncorrelated with each other, as well as the two indicators of stress and control. With regard to cross-comparisons between different sets of variables, the two highest observed correlations, in absolute value, concern reported absenteeism and stress indicator (0.24), and activity indicator and stress indicator (–0.17). The other correlations, being close to zero, are negligible.

**Figure 2:**
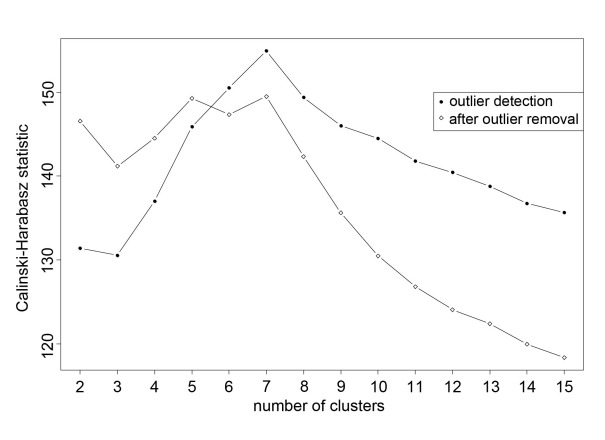
Calinski-Harabasz statistic in k-means clustering with 1000 random starts. First phase (line with circles): detection of outliers; second phase, after outlier removal (line with diamonds): search for optimal number of clusters.

Figure 2 shows values of the CH statistic obtained by repeatedly applying the k-means algorithm with the number of groups increasing from *k* = 2 to *k* = 15. A first run (Figure 2, circles) of this procedure revealed that the partition guaranteeing the maximal separation of groups is that formed by seven clusters (CH = 154.93). Two among these, however, were very small groups, which involved 6 participants in all (6/683, 0.9%), 5 falling in the same cluster plus 1 being isolated. In particular, this latter participant (male) was featured by a very high absenteeism score (300 lost working days, which corresponds to a *z* score of 19.88), a very high stress score (3.27), and a very low activity score (–1.14). Moreover, he was in the 45–54 year age group, was a junior white collar worker, and reported having an organic illness. The other 5 participants (4 males, 1 female) were characterized by the highest scores on alcohol and smoking indicators. In addition, they had the highest scores for stress and absenteeism; 4 of these participants reported a functional illness and the other an organic illness; 3 were in the 45–54 year age group and the other 2 were more than 54 years old; 1 was a blue collar worker, 2 were junior white collar workers, and 2 were senior white collar workers.

As these two small groups constantly kept the same structure while the number of groups increased, we consider them to be formed by outliers, and accordingly we removed them from subsequent analyses. We then restandardized the classification variables on the remaining 677 participants. After outlier removal, correlations between classification variables were practically unchanged. A reduced correlation of reported absenteeism and stress indicator (0.19) is the unique appreciable variation.

The k-means clustering procedure with 1000 random starts, performed under these new circumstances, produced a new set of values on the CH statistic, which we computed, once again, as the number of groups varied from *k* = 2 to *k* = 15 (Figure 2, diamonds). This figure clearly illustrates that the CH statistic assumes the highest values at five clusters (CH = 149.28) and seven clusters (CH = 149.49). After careful consideration we opted for seven clusters, as compared with five (data not shown for simplicity), because this classification better represents the main differences, as well as similarities, between participants.

The first three columns of Table 5 show information about cluster sizes. Numeric cluster labels (first column) are automatically assigned by the clustering procedure, without any specific meaning. The biggest cluster (cluster 5) contains 194 participants (194/677, 28.7%), while the smallest (cluster 7) consists of 20 (20/677, 3%).

**Table 5:** Cluster size and description of subject typologies

Cluster	Count	Percentage	Description of typology	Typology label
1	90	13.3	Highest levels of alcohol habit; mostly nonabsentees, nonsmokers; control indicator highly variable	Alcohol
2	98	14.5	Highest levels of smoking habit; mostly non-physically active, nondrinkers, nonabsentees; control indicator highly variable	Smoking
3	88	13.0	Highest levels of stress; mostly lower levels of control, non-physically active, nonsmokers	High stress
4	57	8.4	Highest levels of physical activity; mostly lower levels of stress, nonsmokers, nondrinkers, nonabsentees	Physical activity
5	194	28.7	Highest levels of control; mostly lower levels of stress, nonsmokers, nonabsentees	High control
6	130	19.2	Lowest levels of stress and control; mostly nonsmokers, nonabsentees	Low stress and control
7	20	3.0	Highest levels of absenteeism; mostly nonsmokers, nondrinkers; stress and control indicators highly variable	Absenteeism
Total	677	100.0		

**Figure 3:**
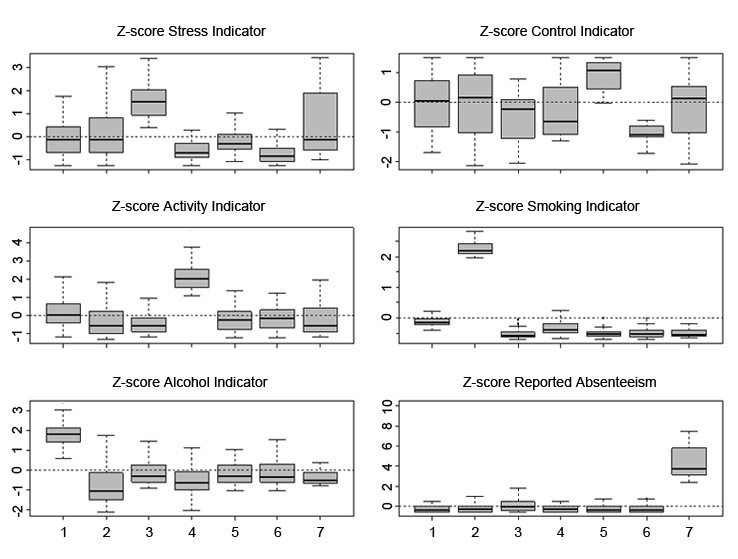
Boxplots of within-cluster distributions of standardized classification variables (x-axis: numeric cluster labels as given in Table 5).

Interpretation of clusters in terms of subject typologies is derived from the analysis of boxplots of within-cluster distributions (Figure 3) of the standardized classification variables. In particular, remarkable associations with specific groups can be observed, such as cluster 3, in which high stress might be associated with low activity, and conversely, a high level of physical activity might be associated with low level of stress in cluster 4. However, similar patterns may not be apparent across the entire survey population, thus suggesting that the relationship between stress and physical activity can assume various forms, especially if considered in the presence of other participants’ characteristics—for example, work category, age, or presence of illnesses.

Differences in absenteeism across clusters are limited, with the exception of cluster 7, which contains all the participants with the greatest number of lost working days. As for stress, nearly 50% of participants in the cluster with the highest levels of stress (cluster 3) reported some absenteeism. The other clusters, including the one with the highest reported activity (cluster 4), show very low levels of absenteeism.

Finally, hypothesis testing procedures, carried out with both parametric and nonparametric methods, empirically supported significant differences between the seven clusters. In detail, the MANOVA procedure led us to reject the null hypothesis of equal cluster centroids with all six classification variables considered simultaneously (Wilks’ lambda: *P* < .001; Hotelling-Lawley trace: *P* < .001). Also, the squared Mahalanobis distance test led us to reject the hypothesis of equality between cluster centroids in all 21 possible comparisons between couples of groups (*P* < .001). Finally, for each variable, univariate ANOVA led us to reject (*P* < .001) the hypothesis of equal cluster means, and Kruskal-Wallis test led us to reject the hypothesis of equal cluster medians (*P* < .001).

The last two columns in Table 5 summarize the findings of cluster characterization in terms of subject typologies. They contain a summary description of specific characteristics of these clusters and the labels of the typologies they represent.

### Relationships Between Subject Typologies and Personal Data, Illness, and Metabolic Syndrome

The final step of our study consisted of assessing potential relationships between subject typologies and each of personal data, illness status, and MeS. This analysis was mostly carried out in descriptive terms through computation and then comparison of within-typology and total percentage distributions. 

**Figure 4:**
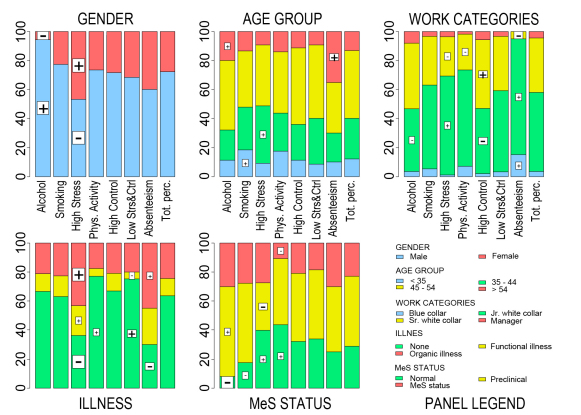
Barcharts of within-typology percentage distributions of personal data (gender, work categories, age group), illness status, and metabolic syndrome (MeS). Details regarding statistical symbols and significance are reported in Multimedia Appendix 1. (Phys = physical, Strs&Ctrl = stress and control; Tot. perc. = total percentage).

Figure 4 presents panels of barcharts of within-typology and total percentages, the latter of which were computed for each variable on the set of 677 participants remaining after outlier deletion, without considering classification. Several worthwhile aspects are detailed in Multimedia Appendix 1, and schematically indicated below. Table 6 summarizes the main features that distinguish the subject typologies in terms of the major (or minor) concentration of participants with certain characteristics as compared with the survey population.

Chi-square test empirically supported significant associations between subject typologies and personal data, illness status, and MeS condition. Specifically, overall associations between subject typologies and either gender, work categories, illness, or MeS status were all significant at the .001 level; association with age group was significant at the .05 level (*P* = .02). The more thorough analysis subsequently carried out with APR (Figure 4, boxed symbols – and +) highlighted that specific associations between single typologies and categories of variables were stronger than expected under statistical independence. In particular, the overall significant relationship between gender and subject typologies appeared to substantially arise from the significant associations involving the alcohol cluster (more males than expected) and the high stress cluster (more females than expected). Moreover, the alcohol cluster included fewer participants without risk factors for MeS and more with preclinical MeS than expected. The high stress cluster turned out to be significantly positively associated with workers aged 35–44 years, junior white collar workers, respondents reporting functional or organic illnesses, and those without MeS. Conversely, the high stress cluster was significantly negatively associated with senior white collar workers. The physical activity cluster was significantly positively associated with participants without illness or without risk factor for MeS. It was significantly negatively associated with senior white collar workers and participants with MeS. The absenteeism cluster proved to be significantly positively associated with older participants (>54 years), blue and junior white collar workers, and participants with organic illnesses. Conversely, it was significantly negatively associated with senior white collar workers and those without illnesses. Finally, the low stress and control cluster was significantly positively associated with participants without illnesses and negatively with those reporting functional illnesses.

**Table 6:** Composition of subject typologies with respect to personal data, illnesses, and metabolic syndrome condition, and prevailing characteristics with respect to the distribution (more or less) of the considered characteristic in the survey population (descriptive analysis)

Typology	Label	Composition
1	Alcohol	More males, senior white collar workers, managers, with preclinical MeS^a^ or MeS, >54 years old
2	Smoking	More <35 years old
3	High stress	More females, junior white collar workers, 35–44 years old, with functional or organic illnesses, without MeS; less blue collar workers, >54 years old, preclinical MeS
4	Physical activity	More blue collar and junior white collar workers, <35 years old, healthy, without MeS; less with functional or organic illnesses, with MeS
5	High control	More senior white collar workers and managers, 45–54 years old
6	Low stress and control	More healthy, 35–54 years old; less <35 years old, with functional or organic illnesses
7	Absenteeism	More females, blue collar and junior white collar workers, >54 years old, with functional or organic illnesses, with MeS; less 35–54 years old; no managers

^a^ Metabolic syndrome.

## Discussion

This study shows the feasibility of assessing health profile, lifestyles, and work habits using an ad hoc self-administered questionnaire via an Intranet application of a large company. The basic ingredients of such an assessment consist in constructing numeric indicators, whichever is the nature of the available information (qualitative and/or quantitative), forming subject typologies from how the indicators combine, and investigating the composition of subject typologies with respect to external variables (ie, variables not involved as classification variables). The main potential of this approach is that it does not require imposing any functional form to relationships between variables, given that, if present, these relationships are learned directly from the data.

### Statistics

A strength of our study is that the use of advanced multivariate statistical methods (ie, PRINCALS [28] and k-means clustering [29] methods) allows the derivation from self-reported data of a series of numeric indicators describing several unobservable variables [34], such as perceived stress and control, and lifestyle domains, as well as the construction of subject typologies and examination of their possible relationship with personal data, illness, and MeS status.

Regarding the specific potentials, unlike statistical–probabilistic modeling, our data-driven approach did not require us to specify functional forms for the relationships between variables, which would have been inappropriate in this context due to the exploratory purposes of the study. In addition, this approach allowed us to get around possible limitations inherent in the available information, especially participants’ self-selection. Being exploratory, these methods do not aspire to generalize findings to sets of subjects not expressly involved in the survey. Their descriptive range is confined within “what is actually observed,” so it does not really matter whether subjects are self-selected or not. Collected data are treated as the unique reference population, whose characteristics are then synthesized, described, and interpreted.

However, in the study we did not completely discard statistical testing procedures. Undoubtedly, the nonidentifiability of the target population implies that test results should be interpreted with some caution, since it is not clear to which population the drawn inferences have to be referred. This is a critical point shared by most surveys, especially Web surveys. Even if the reference population should be a priori well defined (such as in our case, where the reference population is given by the 24,000 workers of the Italian branch of the company), a large number of nonrespondents, typically occurring in these kinds of surveys, would make the set of respondents not representative of the entire population. Nonetheless, statistical tests may help reveal crucial relationships deserving more careful consideration, as well as give rise to new research conjectures which ad hoc studies should address in future investigations. This is the main reason why we have performed inferential analyses as well.

As we claimed, we have performed both parametric and nonparametric testing procedures to take advantage of their specific potentials [29,30]. As it is known in statistical literature, parametric tests may lead to unbiased conclusions, either if data are far from being normally distributed or if any other basic assumption fails to hold, such as the requirement of homogeneity of within-group variances in univariate ANOVA. In most situations the distribution of quantitative data can be rendered approximately normal, or within-group variances can be made homogeneous, by appropriate transformations—for example, by computing the logarithm of values of each variable. However, this procedure may complicate interpretations of results, since these latter have to be referred to transformed, instead of original, data. Conversely, nonparametric procedures, being distribution-free, are not sensitive to departures from normality. They are recognized, however, to be generally less powerful than parametric procedures, in the sense that, for a fixed nominal significance level, nonparametric tests lead to acceptance of the alternative hypothesis when it is true with a lower probability than a parametric test. For these reasons, we have decided to rely on both parametric and nonparametric methods, and then we have considered a test result as “sufficiently revealing” if borne out as significant by both procedures.

### Assessing Stress at Work

Stress is a ubiquitous component of everyday activities, affecting both work and private life. Interest in its assessment has recently increased in view of the tight relationship with a number of negative consequences, either in the subjective domain, such as perceived quality of work and absenteeism, or in the clinical domain, impairing risk profile particularly in the cardiometabolic area [8,35,36]. The majority of stress tools provide metrics that are based on self-reports with standardized questionnaires that are intrinsically prone to bias. These tools are being modified to better focus on stress at work, also in view of the recent policies that, in many countries, mandate stress assessment at work. Usually this approach focuses more on organizational aspects (following motivational models such as the demand-reward [37] or job strain [38]) than on individual physiopathological consequences, such as the increase in sympathetic drive or in hormonal burden (eg, increased cortisol secretion [12]). This selective window might only slightly impair the determination of organizational stress at companywide levels, but may be suboptimal for gathering information useful to designing and planning individualized strategies to tame stress and its health consequences, such as hypertension or worsening metabolic risk. These tools, although simultaneously addressing various aspects of people’s behavior, usually do not employ multivariate statistical techniques, jointly combining ordinal and quantitative data. In previous studies [13-16] we combined information from self-reports focusing on symptom profile and simple indicators of perception of stress, fatigue, and control, with physiopathological data consisting of simple hemodynamics (heart rate and blood pressure) and autonomic indices from heart rate variability. This approach proved valuable to appraise the elevated stress attending a companywide reorganization and to demonstrate the effectiveness of lifestyle strategies to manage stress [13]. In a different study regarding stress management in a clinical setting, in order to curtail the error bias resulting from the inherent imprecision of the subjective measures, we employed a modified approach based on the computation of hidden factors, improving the accuracy of metrics describing a combined stress dimension from multiple indicators [16]. The approach presented in the present study, based on an IT instrument, and on multivariate statistics might prove more robust as a tool to assist individual adherence to self-managed programs for lifestyle improvement and risk reduction, as mandated by several recent guidelines in the hypertension [21] or cardiology area [19].

### Stress and Lifestyle

Because of the large error expected to potentially affect single variables pertaining to ill-defined concepts such as stress, to make allowance for the possible bias of the technique employed (self-reports and unsupervised questionnaire), and to address the multivariate nature of the domains under study (eg, 4SQ, and perceived stress and fatigue scales are expected to be linked together by inextricable interrelationships), we set up indicators of stress, control, and lifestyles, considering in particular activity, smoking, and alcohol habit. In this way it was possible to enhance the information extracted from the data set by synthesizing them in an optimal sense and limiting potentially redundant semantic overlaps. The use of the CH statistic allowed us to select the optimum number of subject typologies that emerged from the analysis and that revolved around few key indicators: alcohol and smoking habits, stress, activity, control, and absenteeism. This approach might thus extend the stress model, which we used for several years in multiple studies involving volunteers or patients [14-16], as well as workers [13], and which provided consistent results, to Web-based self-administered applications.

The present approach evaluates the relationship between typologies and personal data, allowing exploration of key aspects of health promotion and prevention strategies in a normal working population.

For example, stress has been hypothesized as a component or modulator in MeS [36]. In this study, high stress was more prevalent in females, and was observed slightly more in younger participants and junior white collar workers. Thus, stress in this specific population might promote unhealthy behavior, not so much through smoking and poor nutrition, but through inactivity, particularly in the female population [8,39,40]. High stress is also observed more easily in respondents reporting the presence of illness.

Conversely, in the MeS profile, stress unexpectedly showed an elevated percentage of normal. We might interpret this finding to indicate that in the initial phases stress may be perceived subjectively by younger workers, but metabolic implications might require the influence of additional factors over time, such as inactivity favoring the occurrence of obesity, but which cannot be observed in this exploratory investigation. Alcohol abuse could instead play a significant role in facilitating the preclinical condition of MeS. As a final consideration regarding potential validity of the present data, the MeS prevalence in the examined population is similar to that reported for general populations (eg, in the United States [41]).

### Limitations

Because some investigators cast doubts on the validity of self-reported, as compared with non-self-reported, data [11], a few comments seem warranted, particularly considering the specific condition of Web-based applications designed as a part of personalized preventive strategies in the workplace [13]. First, let us consider that every kind of data (Y), either non-self-reported or self-reported, can be conceptualized [11] as the sum of the following factors: “true” data (TD), plus systematic bias (SB) and random error (RE), according to the formula Y= λ_TD_TD + λ_SB_SB + RE, where λ denotes factor loadings.

It should also be noted that even non-self-reported data are not equivalent to error-free data: even simple transcription from paper forms may lead to about a 3% to 26% error rate [42]. Regarding bias, we should consider that it may differ according to the specific context or variable involved—for example, behavioral multicomponent constructs (exercise, stress, etc) may counterintuitively be more accurately represented by self-reports because of the lack of (potentially greater interpretative) bias introduced by a third party (physician, nurse, or technician) [11] and because subjects are likely to better interpret questions about their own behavioral conditions.

Regarding biochemical data, blood pressure, or anthropometrics, we feel confident that only a relative small bias could characterize the self-reported data of this Web-based study, considering that participants were digitally competent and highly motivated to follow the instructions accurately because the usefulness of the final report was contingent on the quality of input data. In this sense, in certain cases when self-reports are the sole source of information, they have been considered “invaluable”, as in the case of the National Health Interview Survey [43]. Moreover, it has been said that Internet-based studies, with a particular focus on self-selection, are of at least as good a quality as those provided by traditional paper-and-pencil methods [44]. For these reasons we avoided putting too much emphasis on single biochemical data, but we combined them with anthropometrics and blood pressure to estimate, as a synthetic descriptor, the MeS status, which, also in our hands [22,27], proved very useful in exploratory population studies.

In short, we are confident that possible errors, if present, are unsystematic, in the sense that they are not in the same direction or with the same magnitude, or else we should suppose that respondents agreed on hindering the survey. For the goal of this study, we therefore considered the constructed indicators and the derived subject typologies to be reliable.

Tools to assess general health and cardiovascular risk, based on a multivariate algorithm, are widely used [45,46], and their main goal is to guesstimate the probability of developing an event in a given time window (usually 5 or 10 years). Conversely, the declared goal of this Web-based questionnaire was to indicate to participants the presence or absence of areas of potential cardiometabolic risk, which could merit a focused improvement, even if the computed global risk was low (as is easily the case for young participants with just one or two usual risk factors). The same applies to those with initial levels of established risk factors, such as prehypertension or nonoptimal lipid profile, frequently combined as forerunners of MeS [46-48]. In this case the use of lifestyle-based interventions could be particularly beneficial, with very low cost and no unwanted effects [19]. The present Internet instrument, although probably suboptimal in providing “hard” health information, might instead be very useful in assisting “soft” lifestyle changes by evoking in participants the awareness and motivation [49] needed to obtain a long-term change in behavior, thus adding an educational flavor to the effectiveness of professional help. Finally, although providing intervention scores to respondents might facilitate compliance, it could also bias the overall data base. Very low occurrence of duplicate responses, however, suggests that this bias was probably minimal.

Additional caution should finally be used in evaluating quantitatively presented data, as other factors, such as the nonsmoking policy of the company, might bias findings, particularly in regard to their external validity.

### Implications for Prevention

In the field of early primary prevention the active role of the individual and the coaching role of the employer have been amply discussed [50]. While the current standard of medical practice for acute conditions relies heavily on institutional resources, prevention must face the challenge of long-term, patient-driven behavioral modifications, based on an agreement on lifestyle determinants. In this model, digital techniques are useful to obtain a streamlined flow of information between patients (or rather people) and the various stakeholders, having employers in the front line of investment. The present investigation showing the feasibility of assessing subject typologies and their relationship with personal characteristics at a workplace with a simple Intranet application might suggest that the time is ripe to test large-scale applications of information and computer technology for better detection and treatment of cardiometabolic risk at the population level, as a complementary benefit offered at the employer’s cost. Studies such as the present one might provide additional momentum to further IT applications as tools for health promotion in the workplace.

## Multimedia Appendix 1

More detailed description of data in Figure 4.

## Abbreviations

4SQ: Somatic Stress Symptoms Score Questionnaire
ANOVA: analysis of variance
APR: adjusted Pearson residual
ATP III: Adult Treatment Panel III
CH: Calinski-Harabasz
IT: information technology
MANOVA: multivariate analysis of variance
MeS: metabolic syndrome
MeTs: metabolic equivalents
PCA: principal component analysis
PRINCALS: nonlinear principal component analysis
VAF: variance accounted for
