# Supplementary material for: Health Promotion in the Workplace: Assessing Stress and Lifestyle With an Intranet Tool
Source: J Med Internet Res. 2011 Nov 8;13(4):e88. doi: 10.2196/jmir.1798 (PMC3222199; doi:10.2196/jmir.1798)
Supplement: Supplementary file 1 [file jmir_v13i4e88_app1.pdf]

Multimedia Appendix 1 [detailed description of data in figure 4]

This appendix reports a more detailed description of several aspects of figure 4. For a better reading we report here the coding of statistical symbols:

Standardized (or adjusted) Pearson residuals (APR), meaning of the symbols - and + and their size inside single bars: observed category frequencies are significantly less (-) or more (+) than expected under the hypothesis of independence. Small + is: 2 ≤ APR < 3, small - is: -3 < APR ≤ -2, each with (1-tailed) significance level: .0013 < *P* ≤ .0228. Medium + is: 3 ≤ APR < 3.5, medium - is: -3.5 < APR ≤ -3, each with (1-tailed) significance level: .0002 < *P* ≤ .0013. Large + is: APR ≥ 3.5, large - is: APR ≤ -3.5, each with (1-tailed) significance level: *P* ≤ .0002.

As indicated in the main manuscript, total percentages are computed for each variable on the set of 677 subjects (here denoted as the “population”) remained after outlier deletion. With regard to gender (Figure 4, 1st panel starting from top left), in cluster 1 (*Alcohol*) there is a higher proportion of males than in the population (94.4% of 90 against the total 72.4% of 677). Females are more highly concentrated in cluster 3 (*High Stress*) and cluster 7 (*Absenteeism*), with percentages, respectively, equal to 46.6% (of 88) and 40% (of 20), against 27.6% of population.

Age group (Figure 4, 2nd panel) examined with respect to subject typologies gives these main results. The youngest subjects (< 35 years) are less concentrated (8.5% of 20) in cluster 7 (*Absenteeism*) and more concentrated (18.4% of 98) in cluster 2 (*Smoking*) than in the population (12%). Subjects with 35 – 44 years are more highly aggregated (39.8% of 88) in cluster 3 (*High Stress*) and less concentrated (20% of 20) in cluster 7 (*Absenteeism*) than overall considered (28.2%). Subjects with 45 – 54 years are more concentrated (52.6% of 194) in cluster 5 (*High Control*) and less concentrated (35% of 20) in cluster 7 (*Absenteeism*) than in the population (46.8%). Finally, the oldest subjects (> 54 years) are much more concentrated (35% of 20) in cluster 7 (*Absenteeism*) and less aggregated in cluster 3 (*High Stress*) and cluster 6 (*Low Stress and Control*) than in the population, with percentages of, respectively, 9.1% (of 88) and 9.2% (of 130) against the total 13%.As for work categories (Figure 4, 3rd panel, top right), cluster 7 (*Absenteeism*) is mostly formed (95% of 20) by blue collars (15% against 3.5% of population) and junior white collars (80% against 54.7% of population); there is one senior white collar only, and no manager is present. Moreover, junior white collars tend to mostly concentrate also in cluster 3 (*High Stress*) and cluster 4 (*Physical Activity*) than in the population, with percentages, respectively, equal to 68.2% (of 88) and 66.7% (of 57) against the total percentage of 54.7%. Clusters 3 and 4 present also lower proportions of senior white collars, with percentages equal to, respectively, 27.3% (of 88) and 24.6% (of 57) against the total of 37.5%. The highest proportions of senior white collars are in cluster 5 (*High Control*) and cluster 1 (*Alcohol*), with percentages equal to, respectively, 47.4% (of 194) and 45.6% (of 90). Finally, managers concentrate more in cluster 1 (7.8% of 90) and cluster 5 (5.7% of 194) than in the population (4.3%), while a lower proportion of managers (1.8% of 57) is in cluster 4 (*Physical Activity*).

As concerns illness status (Figure 4, 4th panel, bottom left), healthy subjects are more aggregated (77.2% of 57) in cluster 4 (*Physical Activity*) and less concentrated (30% of 20) in cluster 7 (*Absenteeism*) than overall considered (63.8%). Subjects with functional illnesses tend

to more highly join together in cluster 7 (*Absenteeism*) and cluster 3 (*High Stress*) than in the population, with respective percentages equal to 25% (of 20) and 20.5% (of 88), against the total of 12%. Conversely, they are less concentrated in cluster 6 (*Low Stress and Control*) and cluster 4 (*Physical Activity*), with percentages respectively of 4.6% (of 130) and 5.3% (of 57). Finally, as expected, subjects with organic illness highly concentrate in cluster 7 (*Absenteeism*), with 45% (of 20), and cluster 3 (*High Stress*), with 43.2% (of 88), than in the population (24.2%). They are less concentrated in cluster 4 (*Physical Activity*), with 17.5% (of 57).

As for MeS (Figure 4, 5th panel, bottom middle), subjects without risk factors for MeS tend to more highly concentrate in cluster 4 (*Physical Activity*), with 43.9% (of 57), and cluster 3 (*High Stress*), with 39.8% (of 88), than in the population (28.8%). The lowest percentages are relative to cluster 1 (*Alcohol*) and cluster 2 (*Smoking*), with respectively the 7.8% (of 90) and 17.3% (of 98). Preclinical subjects are more highly concentrated (62.2% of 90) in cluster 1 (*Alcohol*) and less concentrated (33% of 88) in cluster 3 (*High Stress*) than marginally considered (48.4%). Finally, subjects in MeS condition, which represent the 22.7% of the population, are more highly concentrated in cluster 1 (*Alcohol*) and cluster 7 (*Absenteeism*), in both with 30%, and less represented in cluster 4 (*Physical Activity*), with 10.5% (of 57). The last panel (bottom right) provides color coded legend relevant to the previous data panels.
